# Ten-year surgical experiences with penile cancer at a tertiary care hospital in northwestern Tanzania: a retrospective study of 236 patients

**DOI:** 10.1186/s12957-015-0482-0

**Published:** 2015-02-22

**Authors:** Phillipo L Chalya, Peter F Rambau, Nestory Masalu, Samson Simbila

**Affiliations:** Department of Surgery, Bugando Medical Centre, P.O. Box 1370, Mwanza, Tanzania; Department of Pathology, Catholic University of Health and Allied Science-Bugando, P.O. Box 1464, Mwanza, Tanzania; Department of Oncology, Bugando Medical Centre, P.O. Box 1370, Mwanza, Tanzania; Department of Urology, Bugando Medical Centre, P.O. Box 1370, Mwanza, Tanzania

**Keywords:** Penile cancer, Clinicopathological, Treatment outcome, Tanzania

## Abstract

**Background:**

Penile cancer is an uncommon malignancy in developed countries, but the incidence is as high as 10% to 20% of all male cancers in some developing countries. There is a paucity of published data on this subject in our setting. This study describes the clinicopathological presentation and treatment outcome of this condition in our environment, and highlights challenges associated with the care of these patients and proffers solutions for improved outcome.

**Methods:**

This was a retrospective study of histologically confirmed cases of penile cancer seen at Bugando Medical Centre between January 2004 and December 2013.

**Results:**

There were 236 penile cancer patients representing 2.2% of all male malignancies during the study period. The median age was 47 years with a modal age group of 41 to 50 years. Of the 236 patients, 147 (62.3%) had severe phimosis. The majority of patients (89.8%) were uncircumcised. A history of human papilloma virus (HPV) was reported in 12 (5.1%) cases. One hundred eighty-two (77.1%) patients reported history of cigarette smoking. Seven (6.7%) patients were human immunodeficiency virus (HIV) positive. The majority of the patients (68.6%) presented with Jackson’s stages III and IV. Squamous cell carcinoma was the most common histopathological type (99.2%). Lymph node metastasis was recorded in 65.3% of cases, and it was significantly associated with the tumor size, histopathological subtype, histopathological grade, lympho-vascular invasion, positive resection margins, and urethral involvement (*P* < 0.001). Distant metastasis accounted for 4.2% of cases. The majority of patients (63.1%) underwent partial penectomy. Chemotherapy and radiotherapy were given in 14 (5.9%) and 12 (5.1%) patients, respectively. Complication and mortality rates were 22.0% and 4.2%, respectively. HIV positivity, histopathological stage and grade of the tumor, and presence of metastases at the time of diagnosis were the main predictors of death (*P* < 0.001). The median length of hospitalization was 14 days. Local recurrence was reported in 12 (5.3%) patients. Data on long-term survivals were not available as the majority of patients were lost to follow-up.

**Conclusions:**

Penile cancer is not rare in our environment. The majority of patients present late with advanced stage of the disease. Early detection of primary cancer at an early stage may improve the prognosis.

## Background

Penile cancer is reported to be a rare malignancy in the Western world accounting for less than 1% of adult male cancers [1]. The incidence in Europe and the United States is 0.1 to 0.9 per 100,000 and 0.7 to 0.9 per 100,000 men, respectively [1,2]. However, in some developing countries, the incidence rate of penile cancer is much higher, accounting for up to 10% to 20% of malignant disease in men [2-4]. Incidence also varies according to racial group, ethnicity, and geographical location. Social and cultural habits, hygienic and religious practices interfere significantly with risk factors [5].

The most important etiologic factor of penile cancer is the presence of an intact foreskin. Penile cancer is rarely seen in Jewish individuals, who are circumcised at birth [6]. The risk of this disease in uncircumcised men is approximately threefold higher than that of circumcised men [7]. Other factors associated with increased risk of penile cancer include low socio-economic status, cigarette smoking, human papilloma virus (HPV) infection, lack of penile hygiene, phimosis, and penile inflammation [8].

Although penile cancer may occur at any age, including childhood, it mostly affects the elderly, with a peak incidence around the sixth and seventh decades [9]. While in the Western world penile cancer is a disease of older patients, with most being diagnosed after the age of 50 years [2,9], penile cancer in African population tends to present at a young age with advanced disease and associated poor prognosis [4,10-15].

Patients with cancer of the penis tend to delay seeking medical attention, with 15% to 50% delaying medical attention for more than 1 year from onset [16]. This delay is attributed to embarrassment, guilt, fear, ignorance, and personal neglect. Patients often try to treat themselves with various skin creams and lotions. These may appear to be effective for a time, which further delays the diagnosis and worsens the prognosis. Delays may also attributable to the physician. Some patients with penile cancer report that they receive various salves and antibiotics from their primary care physicians before they see a urologist. A delay in diagnosis and therapy not only affects the likelihood of survival but also limits the ability to retain a functioning and cosmetically satisfactory result [16-18].

The management of penile cancer in resource-limited countries like Tanzania poses major diagnostic and therapeutic challenges which need to be addressed. Late presentation with advanced lesion coupled with lack of diagnostic (such as computed tomography (CT) scan and magnetic resonance imaging (MRI)) and therapeutic facilities such as radiotherapy services are among the hallmarks of the disease in developing countries including Tanzania. The outcome of treatment of penile cancer in most developing countries has been poor because the majority of these patients present late to the hospital with advanced stage. This is partly due to paucity of local data regarding this condition and lack of community awareness on the importance of early reporting to hospital for early diagnosis and treatment. This study was conducted to describe the pattern and treatment outcome of penile cancer patients treated at our center and to highlight challenges associated with the care of these patients and proffer solutions for improved outcome.

## Methods

### Study design and setting

This was a retrospective study of histologically confirmed cases of penile cancer seen at Bugando Medical Centre between January 2004 and December 2013. Bugando Medical Center is a tertiary care and teaching hospital for the Catholic University of Health and Allied Sciences-Bugando (CUHAS-Bugando) in northwestern Tanzania. It has 1,000 beds and serves as a referral center for tertiary specialist care for a catchment population of approximately 13 million people. The hospital has a newly established oncology department which provides care for all patients with histopathologically proven cancers, including penile cancer.

### Study population

The study population included all patients who presented to Bugando Medical Center with histologically confirmed penile cancer during the study period. Patients with incomplete data were excluded from the study. The details of patients were obtained from patients’ files kept in the medical record department, the surgical wards, operating theater, and histopathology laboratory. Information was collected using a preformed questionnaire. Data included in the questionnaire were age of the patient, risk factors for penile cancer, clinical presentation, anatomical site, tumor stage, histopathological type and grade, presence of lymph node or distant metastases, treatment modalities, treatment outcome (that is, postoperative complications, length of hospital stay, and mortality), and follow-up. The clinical stage of the disease was assigned to each patient using the Jackson Classification for Cancer of the Penis [19] as follows: stage I (confined to glans or prepuce), stage II (invasion into shaft or corpora), stage III (operable inguinal lymph node metastasis), and stage IV (tumor invades adjacent structures, inoperable inguinal lymph node metastasis).

### Statistical data analysis

The statistical analysis was performed using the Statistical Package for Social Sciences (SPSS) version 17.0 for Windows (SPSS, Chicago, IL, USA). The median (and IQR) and ranges were calculated for continuous variables, whereas proportions and frequency tables were used to summarize categorical variables. The chi-square (*χ*^2^) test was used to test for the significance of association between the independent (predictor) and dependent (outcome) variables in the categorical variables. The level of significance was considered as *P* < 0.05. Multivariate logistic regression analysis was used to determine predictor variables that predicted the outcome.

### Ethical consideration

Ethical approval to conduct the study was obtained from the CUHAS-Bugando/BMC joint institutional ethic review committee before the commencement of the study.

## Results

### Socio-demographic profile

During the study period, a total of 11,345 male malignancies were registered at Bugando Medical Centre. Of these, 248 (2.2%) were histopathologically confirmed penile cancer (Table 1). Out of these, 12 patients were excluded from the study due to missing data. Thus, 236 patients were enrolled into the study. The age of patients at diagnosis ranged from 21 to 78 years with the median age of 47 years (IQR = 45 to 49 years). The peak age incidence was in the 41- to 50-year age group accounting for 43.6% of cases. One hundred thirty-three (56.4%) patients were younger than 50 years of age. All of our patients were blacks of African descent. The majority of them (204 (86.4%)) came from the rural areas located a considerable distance from Mwanza City, and most of them (212 (89.8%)) had either primary or no formal education. More than 80% of patients were unemployed, and the vast majority of these patients (220 (93.2%)) had no identified health insurance.

**Table 1:** **Annual distribution of patients (** ***N*** **= 248)**

**Year at presentation**	**Number of patients**	**Percentages**
2013	24	9.7
2012	28	11.3
2011	32	12.9
2010	27	10.9
2009	30	12.1
2008	18	7.3
2007	25	10.1
2006	29	11.7
2005	16	6.5
2004	19	7.7

### Etiological risk factors

Of the 236 patients with penile cancer, 147 (62.3%) had severe phimosis not allowing the possibility of exposing the glans. The majority of patients (212 (89.8%)) were uncircumcised and only 24 (10.2%) patients had circumcision. Of these 24 patients who had circumcision, only 2 (8.3%) underwent the procedure during the neonatal period, and in the remaining 22 (91.7%) patients, the procedure was performed during adolescence and in adulthood. HPV infection was reported in 12 (5.1%) cases. One hundred and eighty-two (77.1%) patients reported history of cigarette smoking. The number of cigarettes per day that the patients consumed tobacco was not reported, nor was the length of time those patients had been tobacco smokers preventing further statistical analysis to be conducted. One (0.4%) patient developed penile cancer from a penile human bite scar (Marjolin’s ulcer). This patient sustained penile human bite injury during oral sexual intercourse. He was treated successfully and got cured. Seven months later, a malignant ulcer on the healed penile scar developed, which was confirmed histopathologically as squamous cell carcinoma. Fifty-four (22.9%) patients reported history of multiple sexual partners, and most of them had suffered sexually transmitted diseases once or several times in their adulthood. Human immunodeficiency virus (HIV) status was known in 104 patients. Out of the 104 patients with known serostatus, 7 (6.7%) were reported to be HIV positive.

### Clinicopathological characteristics among patients with penile cancer at Bugando Medical Centre

The duration of symptoms at presentation ranged from 2 months to 7 years with a median duration of 22 months, and the majority of patients (164 (69.5%)) presented between 1 and 5 years of onset of illness. Fifty-two (22.0%) and 20 (8.5%) patients presented within 1 year and after 5 years of onset of illness, respectively. Two hundred eleven (89.4%) patients had undergone some form of intervention in peripheral dispensaries and hospitals before being referred, with dressing, antibiotics, and inadequate surgical resection being the most common intervention and some of them were treated as cases of syphilis. Anatomical site, Jackson’s stage, tumor size, histopathological type/grade, and metastases at the time of diagnosis are shown in Table 2. Regarding the histopathological type, the classic squamous cell carcinoma (SCC) was the predominant subtype seen in 199 (85.0%) patients. This was followed by basaloid subtype in 12 (5.1%), verrucous subtypes in 5 (2.1%), and sarcomatoid in 2 (0.9%) patients. SCC subtype was not documented in 18 (7.6%) patients. Lymph node metastasis at the time of diagnosis was significantly associated with anatomical site (OR = 3.4, 95% CI (2.1 to 9.8), *P* = 0.002), tumor size (OR = 2.9, 95% CI (1.1 to 6.4), *P* = 0.011), histopathological subtype (OR = 6.2, 95% CI (3.1 to 8.6), *P* = 0.001), histopathological grade (OR = 4.0, 95% CI (2.2 to 5.6), *P* = 0.003), lympho-vascular invasion (OR = 2.7, 95% CI (1.8 to 5.4), *P* = 0.032), positive resection margins (OR = 3.8, 95% CI (2.6 to 7.1), *P* = 0.012), and urethral involvement (OR = 4.3, 95% CI (2.1 to 6.0), *P* = 0.000) in multivariate logistic regression analysis. Distant metastasis occurred mainly to the liver, lung, bone, and brain in 5 (50.0%), 3 (30.0%), and 1 (10.0%) each, respectively.

**Table 2:** **Anatomical site, Jackson’s stage, tumor size, histopathological type/grade, and metastasis**

**Variable**	**Response**	**Frequency**	**Percentages**
Anatomical site	Glans penis	142	60.1
	Glans + shaft	32	13.6
	Prepuce only	18	7.6
	Glans + prepuce	18	7.6
	Shaft + prepuce	14	5.9
	Shaft only	10	4.2
	Whole penis + scrotum	2	0.8
Histopathological type	Squamous cell carcinoma	234	99.2
	Soft tissue sarcoma	1	0.4
	Small-cell carcinoma	1	0.4
Tumor size	Less than 2 cm	56	23.7
	2 cm or more	180	76.3
Histopathological grade	Well differentiated	143	60.6
	Moderately differentiated	52	22.0
	Undifferentiated (anaplastic)	31	13.1
	Not documented	10	4.2
Jackson’s classification stage	I	24	10.2
	II	26	11.0
	III	132	55.9
	IV	30	12.7
	Not documented	24	10.2
Metastasis at the time of diagnosis	Lymph node metastasis	154	65.3
	Distant metastasis	10	4.2
	No evidence of metastasis	72	30.5

### Radiological and laboratory investigations

Chest X-rays performed in all (100%) patients confirmed lung metastasis in three (1.3%) patients. Skeletal survey revealed bony metastasis in one patient. Abdomino-pelvic ultrasound performed in 162 (68.6%) patients detected pelvic (iliac) lymph nodes and liver metastases in 84 (51.9%) and 5 (3.1%) patients, respectively. No patient had a CT scan or MRI examination due to the lack of these imaging facilities at our center. Liver function tests were performed in all patients and revealed abnormal results in ten (4.2%) patients. HIV status was known in 104 patients and revealed positive results in 7 (6.7%) patients. The CD4+ count among HIV-positive patients was available in only four patients and ranged from 126 to 712 cells/μl with a median of 234 cells/μl (IQR = 230 to 238 cells/μl). VDRL test performed in 98 (41.5%) patients revealed negative results in all patients.

### Treatment modalities

**Figure 1:**
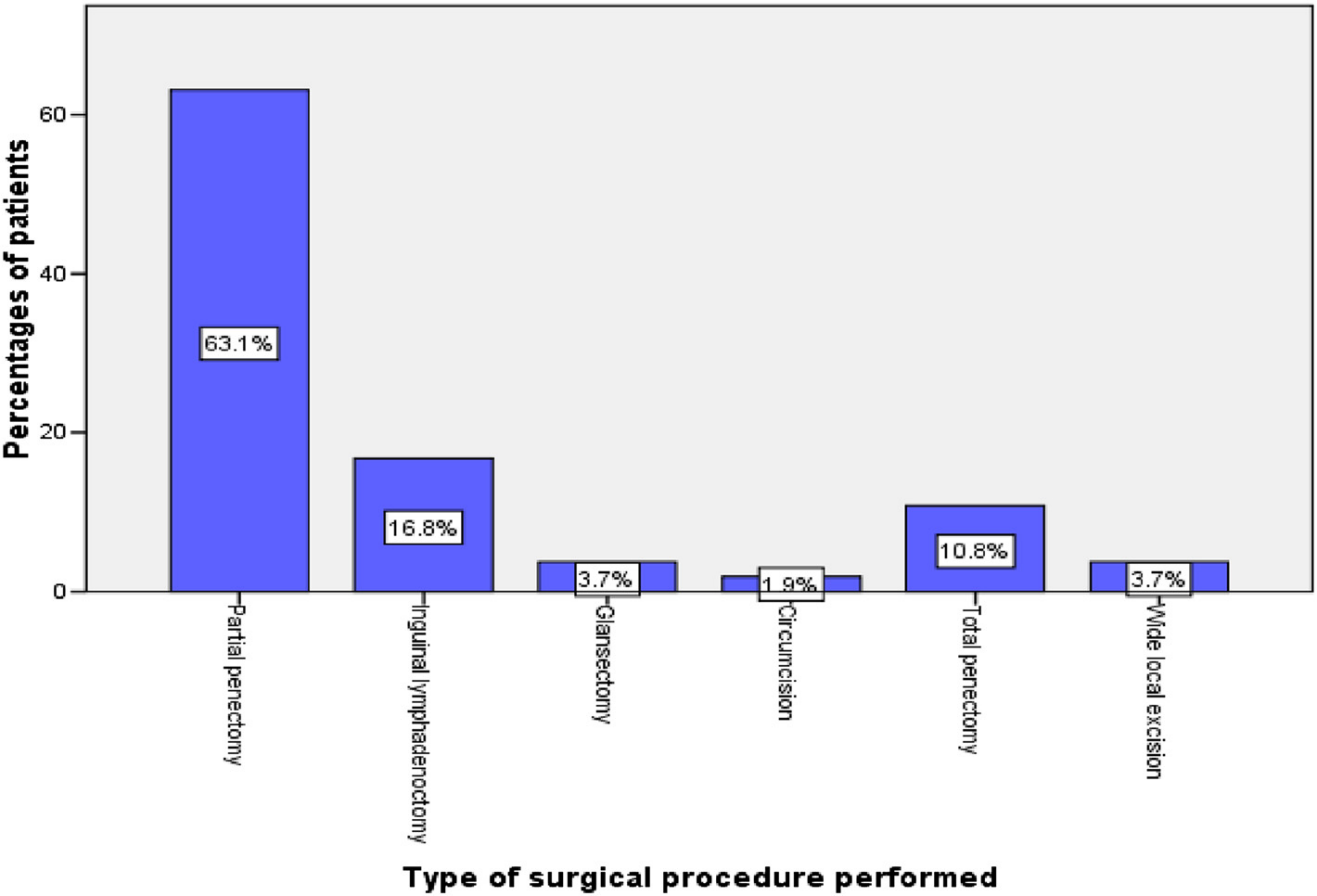
**Distribution of patients according to the type of surgical procedures performed.**

Out of 236 patients, 234 (99.2%) underwent 268 surgical procedures for penile cancer, and the remaining 2 (0.8%) patients were unfit for surgery. Of the type of surgical procedures performed, partial penectomy was the most frequently performed surgical procedure, accounting for 63.1% of cases (Figure 1). Inguinal lymphadenectomy was performed in only 16.8% of cases. No patient had pelvic lymphadenectomy. The use of chemotherapy was documented in 14 (5.9%) patients. Of these, 1 (7.1%) was given chemotherapy as a neo-adjuvant therapy, whereas in the remaining 13 (92.9%) patients, chemotherapy was used as adjuvant therapy. Cisplatin in combination with other cytotoxic agents such as bleomycin and methotrexate was the most commonly used drug. Radiotherapy was used as adjuvant therapy in only 12 (5.1%) patients.

### Clinical outcome and follow-up of patients

A total of 58 postoperative complications were recorded in 52 patients giving a complication rate of 22.0%. Of these, surgical site infection (SSI) was the most common postoperative complication accounting for 44.8% of cases (Table 2). On multivariate logistic regression analysis, the rate of SSI was significantly high in HIV-positive patients as compared with HIV-negative patients (*P* = 0.003). The overall length of hospital stay (LOS) ranged from 5 to 34 days with a median of 14 days (IQR = 12 to 16 days. Patients who developed postoperative complications stayed longer in the hospital, and this was statistically significant (*P* = 0.012). Out of 236 patients, 226 (95.8%) were discharged alive. Ten patients died in the hospital giving a mortality rate of 4.2%. According to multivariate logistic regression analysis, HIV positivity (OR = 4.4, 95% CI (2.3 to 8.7), *P* = 0.011), histopathological stage (OR = 5.2, 95% CI (2.7 to 8.8), *P* = 0.004) and grade of the tumor (OR = 3.3, 95% CI (1.3 to 5.1), *P* = 0.03), and presence of metastases at the time of diagnosis (OR = 5.6, 95% CI (3.4 to 8.9), *P* = 0.001) were the main predictors of death.

Follow-up of patients among survivors ranged from 3 to 61 months with a median of 22 months (IQR = 20 to 24 months). At the end of 5 years, only 54 (23.9%) patients (survivors) were available for follow-up. Cancer recurrence was reported in 12 (5.3%) patients who had surgical treatment. All cases of cancer recurrence occurred from 6 months to 1 year (median = 8 months) after definitive treatment. Tumor size (OR = 1.6, 95% CI (1.1 to 5.0), *P* = 0.014), histological grade of the tumor (OR = 2.3, 95% CI (1.2 to 3.9), *P* = 0.001), stage of the tumor (OR = 4.2, 95% CI (1.3 to 6.8), *P* = 0.023), and presence of metastasis at the time of diagnosis (OR = 2.7, 95% CI (1.8 to 6.8), *P* = 0.002) were the main predictors of cancer recurrence in multivariate logistic regression analysis. Data on long-term survivals were not available as the majority of patients were lost to follow-up.

## Discussion

Cancer of the penis, though rare in developed countries, constitutes a continuing challenge to urologist and oncologists practicing in developing countries where the frequency of this disease is as high as 10% to 20% of all male cancers [2-4]. In this review, penile cancer accounted for 2.2% of all diagnosed male malignancies seen during the study period in our setting. This figure is higher than 0.1% that was reported by Magoha and Ngumi [15] in Kenya. Favorito *et al.* [9] reported a high figure of 5.7% in northeastern Brazil. There is a marked variation in the incidence of penile cancer worldwide, with western countries having a low rate of less than 1% compared to higher rates reported in many countries in Africa, Asia, and North America [2-4]. These differences in incidence rates reflect differences in risk factors for penile cancer between the study settings.

Penile cancer is typically a disease of older men and rates increase steadily with age [9]. Many studies in developed countries indicate a higher incidence of the disease between the sixth and seventh decades of life [9,14,15]. As reported in other African studies [16,17], the median age of 47 years in this study was younger than the age described in most developed countries; about 10 years difference has been reported in these studies [17]. In this study, 56.4% of patients were found to be younger than 50 years of age in their fourth and fifth decades of life. We could not found in the literature the reasons for this age differences, and this needs to be investigated further.

In keeping with other studies performed in developing countries [14,15], the majority of patients in this series came from the rural areas located a considerable distance from the study area, and more than 85% had either primary or no formal education. This observation has an implication on accessibility to healthcare facilities and awareness of the disease.

Phimosis is considered an important risk factor for the development of penile cancer and is found in approximately 25% to 75% of patients with this cancer in the largest series [8]. It has been proposed that inadequate hygiene of the preputial sac with consequent accumulation of smegma leads to a chronic local inflammatory process, contributing to the genesis of penile cancer [20]. In our study, more than 60% of patients presented with severe phimosis not allowing the possibility of exposing the glans. The majority of patients (89.2%) in this study were uncircumcised. This is in conformity with numerous previous reports that indicate penile cancer to be common among the uncircumcised males [6,8,10-13,15]. Neonatal circumcision as practised by the Jews is known to prevent the development of cancer of the penis, and this is largely mediated by prevention of phimosis [6,15,20]. However, circumcision in adolescence and in adults does not prevent the development of penile cancer [20]. In our study, only 2 (8.3%) patients underwent the procedure during the neonatal period, and in 22 (91.7%) patients, the procedure was performed during adolescence and in adulthood. Uncircumcised men are three times more likely to develop this form of cancer than men who are circumcised [6,20]. In addition, men who have an unretractable foreskin are ten times more likely than other men to develop penile cancer [20].

HPV has been reported to be a risk factor in the development of penile cancer [21]. Many studies have shown the presence of HPV types 16 and 18 in penile cancer [21-23]. The mechanism by which HPV leads to malignant transformation is likely mediated through two viral genes, E6 and E7, which are actively transcribed in HPV-infected cells. The E6 and E7 proteins bind to and inactivate the host cell’s tumor suppressor gene products p53 and pRb (retinoblastoma gene), both of which are known negative regulators of cellular proliferation, leading to uncontrolled growth [22]. The prevalence of HPV infection in penile carcinoma ranges widely, from 15% to 71% in different studies [23]. In the current study, HPV infection was reported in only 5.1% of cases, a figure which is significantly lower than that reported in the literature [23]. The reason for the low rate of HPV in our study may be explained by the fact that patients with penile cancer in our setting are not routinely screened for HPV infection.

Several studies have shown an association between penile cancer and smoking. Hellberg *et al.* [24] found a relationship between penile cancer and smoking that was direct, dose-related, and independent of other known risk factors. Harish and Ravi [25] extended these observations by demonstrating that the consumption of products made from tobacco is also related to the incidence of penile cancer independent of other factors. In our series, we observed a predominance of smokers, representing 77.1% of cases. This study, despite not having used a control group, showed that more than three quarter of the patients with cancer of the penis were smokers, suggesting that smoking may represent a risk factor for the development of penile cancer. The association between smoking and penile cancer can be explained by the fact that tobacco products (nicotine) usually become concentrated in smegma, making it carcinogenic, especially in men with phimosis.

Injury to the penis had been reported to increase the risk of penile cancer [8]. In the present study, we had one patient who presented with an early appearance of penile cancer (Marjolin’s ulcer) developing in a penile human bite scar. Marjolin's ulcer is a rare and often aggressive cutaneous malignancy that arises in previously traumatized, degenerated, and chronically inflamed skin or scar tissue [26]. Marjolin’s ulcers are commonly mistaken for an infected ulceration occurring at the scar tissue sites and may often be overlooked. In our study, the majority of penile cancers were mistaken for syphilis and were treated with dressing and antibiotics with no improvement. A high index of suspicion is therefore required in the management of chronic non-healing penile ulcers that are recalcitrant to therapy, and all suspected lesions should be biopsied.

Multiple sexual partners and sexually transmitted diseases especially genital warts have been reported to increased the risk of developing penile cancer [8]. In the current study, more than 20% of patients reported history of multiple sexual partners, and most of them had suffered sexually transmitted diseases once or several times in their adulthood. Many studies have shown an association between penile cancer and a history of multiple sexual partners and early age of first intercourse [6].

Infection with HIV, the virus that causes acquired immune deficiency syndrome (AIDS), is a risk factor for penile cancer. When a person is HIV positive, their immune system is less able to fight off early-stage cancer [6,27]. Penile cancer has been observed in HIV-positive men and in transplant recipients, who carry a 36-fold increase of the risk of cancer development at any site [27,28]. Although the AIDS epidemic has not led to an increased incidence of penile cancer in areas such as Uganda, where HIV infection is widespread, however, there has been a significantly increased incidence of penile among HIV patients, and immune suppression is considered a relevant predisposing factor to penile cancer [28,29]. In our study, HIV status was known in 104 patients, out of whom 7 (6.7%) were reported to be HIV positive. Men with AIDS have a higher risk of developing penile cancer. This higher risk seems to be related to their lowered immune response, but lifestyle factors may also play a role. In some studies, men with penile cancer who were HIV positive were more likely to smoke and to be infected with HPV than HIV-negative men with penile cancer [6,27].

The majority of patients in this study presented late with an advanced stage of the disease (Jackson’s stages III and IV) with operable or inoperable inguinal metastases and tumors involving adjacent structures or distant metastases which is in keeping with other studies performed in developing countries [9,15]. This finding is of concern because it is well established that the advanced stage is strongly correlated with the degree of invasion and probability of regional and systemic metastases suggesting a worse prognosis for these patients [30]. The reason for this late presentation of penile cancer in this locality is due to the delay in seeking appropriate medical advice as a result of socio-cultural taboos and ignorance. The late presentation of cases is an area of cancer care in our center that requires urgent attention. Detecting primary cancer at an early stage contributes to improved chances for successful treatment and thus for survival.

Cancer of the penis is usually an epidermoid tumor arising from the glans penis or the mucosal lining of the prepuce [15]. In this study, the glans penis was the most frequent anatomical site involved in 60.1% of cases. This observation concurs with other studies [9,15] which reported the glans penis as the most frequent anatomical site involved in penile cancer.

The presence of metastases in the regional lymph nodes is the main factor predicting an unfavorable prognosis for patients with penile cancer [18,30]. In the present study, lymph node metastasis at the time of diagnosis was recorded in 65.3% of cases, and it was significantly associated with anatomical site, tumor size, histopathological subtype, histopathological grade, lympho-vascular invasion, positive resection margins, and urethral involvement. A similar observation was reported by others [15,18,28]. High lymph node metastasis in our study is due to the fact that most patients in the present study present late when the disease is already in advanced stages. In developing countries like ours, especially in rural areas with poor living conditions most patients are already in advanced stages of disease at the time of diagnosis of penile cancer which has been proven both in the present study and in the literature [9,15].

Metastatic spread to distant sites (lungs, liver, bone, and brain) is uncommon and is reported to occur in 1% to 10% of cases in most large series. Such metastases usually occur late in the course of the disease after the local lesion has been treated [28]. Distant metastases in the absence of regional node metastases are unusual [18,28]. In our series, distant metastasis was reported in 4.2% of cases and occurred mainly to the liver, lung, bone, and brain. Low rate of distant metastasis in this study may be attributed to by the lack of advanced staging diagnostic facilities (such as CT scan, MRI, and positron emission tomography) in our setting.

The commonest histopathological type in penile cancer is squamous cell carcinoma occurring in more than 95% of cases [10,11,13,15]. This finding is in agreement with the present study in which more than 99% of penile cancers were squamous cell carcinoma. Other histological types about 5 in 100 penile cancers (5%) are adenocarcinomas, melanoma, soft tissue sarcomas, urethral tumors, lymphomas, basal cell carcinomas, and metastases; these types are extremely rare [31].

The treatment modalities of penile cancer include surgery, chemotherapy, and radiotherapy [13,15,32]. Surgery is considered to be the mainstay of treatment of penile cancer, with many early-stage patients being cured with surgery alone [32]. The goals of surgical treatment of penile cancer include the complete resection of the primary tumor, with negative margins to reduce the risk of local recurrences, and pathologic staging of the tumor and inguinal lymph nodes to provide necessary prognostic information [30]. In keeping with other studies [13,15], surgery was the most common modality of penile cancer treatment in this study. Of the type of surgical procedures performed, partial penectomy was the most frequently performed surgical procedure, accounting for 63.1% of cases. This observation concurs with what was reported by Magoha and Ngumi [15] in Kenya. In this study, inguinal lympadenectomy was performed in 16.8% of cases after penectomy, a figure which is higher than that reported in Kenya by Magoha and Ngumi [15]. The overall prognosis in all stages of penile cancer is known to depend largely upon the presence or absence of inguinal lymph node metastases [30].

Recent advances in surgical therapy of penile cancer including Mohs micrographic surgery, laser therapy, and reconstructive surgery can provide satisfactory surgical treatment while minimizing functional loss of the penis and avoiding the considerable complications associated with radiotherapy [33,34]. However, none of our patients had this form of treatment due to lack of facilities for these treatment modalities at our center.

Chemotherapy has been utilized in the treatment of advanced penile cancer either alone or in combination with surgery or radiotherapy. Bleomycin, cisplastin, and methotrexate are the most effective, but complete responses are rare [35]. In the present study, only 5.9% of patients received chemotherapy and more than two thirds among those offered chemotherapy defaulted and did not complete the course. We could not establish the reasons for non-adherence to chemotherapy in our study due to the retrospective nature of the study.

Radiotherapy treatment either alone or in combination with surgery or chemotherapy has been shown to improve local control rates. However, in our study, only 5.1% of patients who required radiotherapy had access to this form of treatment. This concurs with other studies in resource-limited countries [11-13,15]. Failure to access this modality of treatment in our patients can be explained by the fact that radiotherapy is not available in our center, and therefore, patients requiring this form of treatment had to travel long distances to receive radiotherapy at the oncological center. This sad observation calls for urgent establishment of radiotherapy services in our center.

In this study, the postoperative complication rate was 22.0%, which is a higher rate than that reported by other authors [13,15]. Surgical wound infection was the most common complication but was usually superficial and easily controlled by local wound care. Additional operations were required in nine (6.7%) patients who developed postoperative complications, such as wound dehiscence, Fournier’s gangrene, and urethral stricture (Table 3). These required additional operations were associated with prolonged hospital stay and higher cost of treatment. The median duration of hospital stay in our study was 14 days, which is higher than that reported in other studies [11,13,15]. This can be explained by the high rate of postoperative complications which required additional operation care and, subsequently, prolonged hospital stay.

**Table 3:** **Distribution of patients according to postoperative complication**

**Postoperative complication**	**Frequency**	**Percentages**
Surgical site infections	26	44.8
Deep venous thrombosis (DVT)	9	15.5
Chronic pain	8	13.8
Scrotal edema	6	10.3
Wound dehiscence	4	6.9
Fournier’s gangrene	3	5.2
Urethral stricture	2	3.4

Our overall mortality rate in the present study was 4.2% that is relatively lower than that reported in other studies [15,35]. In the present study, mortality rate was significantly high in HIV patients and in patients with high stage and grade of the tumor and those who had metastases and local recurrences.

In the present study, local recurrence was recorded in 5.3% of cases which is a higher rate than that reported by other authors [35,36]. Tumor size, histological grade of the tumor, stage of the tumor, and presence of metastasis at the time of diagnosis were the main predictors of local recurrence.

The follow-up of patients in this study was generally poor, and data on long-term survival were not available. This observation concurs with other reports done in developing countries [15,35]. Poor follow-up of patients in our study may be explained by the fact that the majority of patients were lost to follow-up at the end of the study period. The potential limitation of this study is the fact that the information about some patients was incomplete in view of the retrospective nature of the study, and this might have introduced some bias in our findings. Also, this study included only patients who were evaluated and treated at a single institution, which may not reflect the whole population in this region, despite the fact that approximately 70% of oncologic patients in northwestern Tanzania are managed at our center. However, despite this limitation, the study has provided local data that can help healthcare providers in the management of patients with penile cancer. The challenges identified in the management of penile cancer in our setting need to be addressed in order to deliver optimal care for these patients.

## Conclusions

Penile cancer is not rare in our environment affecting mostly young adult males in their fourth decades of life, uncircumcised with history of smoking and sexually transmitted diseases including HIV. The majority of patients present late with an advanced stage of the disease and a high rate of inguinal lymph node metastasis. Late presentation, poor accessibility to healthcare facilities, lack of advanced diagnostic and therapeutic facilities, lack of radiotherapy services at our center, and lost to follow-up are among the hallmarks of the disease in this region and pose a great challenge in the management of these patients. Early recognition and aggressive treatment of penile cancer and close follow-up are urgently needed to improve outcomes in our environment. Health education is highly needed to discourage patients with penile cancer from presenting late to hospital when the disease is in its advanced stage. A high index of suspicion is required in the management of chronic non-healing penile ulcers that are recalcitrant to therapy and all suspected lesions should be biopsied. In addition, encouraging neonatal circumcision will go a long way towards reducing the incidence of penile cancer in later life. Establishment of radiotherapy services at our center is highly recommended.

## Abbreviations

BMC: Bugando Medical Centre
CT: computed tomography
CUHAS: Catholic University of Health and Allied Sciences
HIV: human immunodeficiency virus
HPV: human papilloma virus
IQR: interquartile range
MRI: magnetic resonance imaging
SCC: squamous cell carcinoma
SPSS: Statistical Package for Social Sciences.
